# Hypoxia Induces Growth Differentiation Factor 15 to Promote the Metastasis of Colorectal Cancer via PERK-eIF2*α* Signaling

**DOI:** 10.1155/2020/5958272

**Published:** 2020-01-27

**Authors:** Hongtu Zheng, Yuchen Wu, Tianan Guo, Fangqi Liu, Ye Xu, Sanjun Cai

**Affiliations:** ^1^Department of Colorectal Surgery, Fudan University Shanghai Cancer Center, Shanghai, China; ^2^Department of Oncology, Shanghai Medical College, Fudan University, Shanghai, China

## Abstract

Hypoxia plays an essential role in orchestrating Epithelial-mesenchymal transition and promoting metastasis of colorectal cancer. However, the underlying mechanisms are still not well elucidated. Here, we present that hypoxic exposure causes endoplasmic reticulum stress and activates the unfolded protein response pathways, which drives GDF15 expression in colorectal cancer cells. Mechanistically, upregulated CHOP led by activated PERK-eIF2*α* signaling promotes GDF15 transcription via directly binding to its promoter. Further study implicates that hypoxia-induced GDF15 is required for the EMT and invasion of colorectal cancer cells; enforced expression of GDF15 promotes the mitochondrial oxidation of fatty acids in colorectal cancer cells. Moreover, the abrogation of GDF15 results in smaller xenograft tumors in size and impaired metastasis. *GDF15* is expressed much more in tumor tissues of CRC patients and displays positive correlations with *CHOP* and HIF1*α* in mRNA levels. Our study demonstrates a novel molecular mechanism underlying hypoxia-promoted metastasis of CRC and provides PERK signaling-regulated GDF15 as a new and promising therapeutic target for clinical treatment and drug discovery.

## 1. Introduction

As one of the most malignant cancers, colorectal carcinoma (CRC) is the second most prevalent cause of death from cancer in the western world. About one-third of the 147,000 patients of CRC will succumb to the disease each year in the USA [1].

With the rapid and progressive growth of malignant tumors (e.g., CRC), tumor cells in the ischemic/hypoxic condition are prone to transform to the metastatic phenotype with reduced intercellular adhesion as well as increased cell motility and invasiveness [2–5]. Hypoxia or the overexpression of Hypoxia-induced factor (HIF) could sufficiently induce epithelial-mesenchyme transition (EMT) and invasion in multiple cell types, including CRC [5–7]. Through regulating distinct signal transduction pathways, hypoxia orchestrates EMT of various cancers in direct or indirect manners [7–9]. However, the detailed molecular mechanisms underlying hypoxia-mediating metastasis remain obscure.

The unfolded protein response (UPR) acts as a cytoprotective response to ER stress led by the accumulation of unfolded or misfolded proteins inside of this organelle [10–12]. In mammalian cells, three core UPR-associated signaling pathways orchestrate the adaptive responses to alleviate ER stress, including the expansion of the ER capacity, promotion of ER-associated degradation and chaperone functions, and apoptosis if unabated [13]. Sustained ER stress and activation of UPR pathways have been observed in many cancers and are crucial in tumor progression [14–18]. Hypoxic cells in a tumor mass accumulate free radicals resulted from shift cellular metabolism and also stress the ER to accumulate misfolded proteins, which could activate the UPR pathways [19]. However, it is still uncertain whether hypoxic condition induces the activation of UPR in CRC cells. Growing studies have reported that ER stress could promote EMT and invasion in types of cancers [20–22]. PERK-eIF2*α* branch is revealed as critical signaling in regulating EMT of tumor cells [21]. However, it remains unclear that the downstream molecular of PERK-eIF2*α* branch is involved in the regulation of EMT. New mediators are needed to be characterized to clarify the molecular mechanism underlying UPR-associated tumor metastasis.

Growth differentiation factor 15 (GDF15), also known as macrophage inhibitor cytokine (MIC-1), is a member of the transforming growth factor-*β* (TGF-*β*)/bone morphogenetic protein (BMP) superfamily and a secreted protein circulating in plasma as a 25 kDa homodimer [23, 24]. GDF15 has been reported to have multiple functions in various pathologies, including inflammation, cancer, cardiovascular diseases, and obesity [25]. Moreover, the circulating levels of GDF15 are elevated in several cancers, including endometrial cancer, prostate cancer, pancreatic cancer, and colorectal cancer [26–29]. However, the exact role of GDF15 in tumor progression still needs to be well elucidated.

Previous studies uncovered that CCAAT/enhancer-binding protein homologous protein (CHOP), a critical transcription factor in the downstream of PERK-eIF2*α* signaling, drives the transcription of GDF15 in murine skeletal muscle upon metabolic stress [30]. In this study, we present that ER stress is dramatically induced by hypoxia exposure and subsequently activated PERK-eIF2*α* signaling promotes the metastasis via regulating GDF15 expression in CRC cells.

## 2. Materials and Methods

### 2.1. Cell Culture and Human Tissue Samples

Human colorectal cancer cell lines, HT29 and SW480, were obtained from Cell Bank of Shanghai, Shanghai Institutes of Biological Sciences, Chinese Academy of Sciences. HT29 cells were P141 when purchased and SW480 were P108. The cells used in this study were passaged within 10 generations. HT29 cells were cultured in high-glucose Dulbecco's modified Eagle medium (DMEM; Corning, #MT10013CV) supplemented with 10% v/v fetal bovine serum (Gibico, Thermo Fisher, #26140). SW480 cells were cultured in Leibovitz's L-15 medium (Invitrogen, #11415064) supplemented with 10% v/v FBS (Gibico, Thermo Fisher, #26140). For cell experiments, the cells were resuspended in the medium for 1 × 10^5^/ml and seeded in the plate according to the assay. For TUDCA treatment, sodium TUDCA (Sigma, #T0266) with the purity (TC) of >95% was dissolved in ethanol for a stock concentration of 40 mmol/L. Ethanol was added into the medium for vehicle control.

For the normoxia group, cells were grown at 37°C in a humidified atmosphere of 5% CO_2_ (∼20% O_2_) as previously described [31]. For hypoxia exposure (1% O_2_), cells were cultured in the hypoxia chamber (5% CO_2_/94% N_2_, Forma Scientific, Marietta, OH, USA) for the indicated time.

Tissue samples of 21 CRC patients were collected from Fudan University Shanghai Cancer Center (Shanghai, China) between December 2009 and June 2016. The study is approved by the ethics committee of Fudan University Shanghai Cancer Center and carried out in accordance with Ethical Principles for Medical Research involving Human Subjects (World Medical Association Declaration of Helsinki). The experiments were undertaken with the understanding and written consent of each subject.

### 2.2. Quantitative Real-Time PCR

qRT-PCR was conducted as previously described [32]. Briefly, cell or tissue samples were fixed and lysed by TRizol (Invitrogen, Carlsbad, CA, USA) and then prepared for total RNA extraction. Total RNA was then used for cDNA synthesis by using M-MLV reverse transcriptase following the manufacturer's instructions. Indicated mRNA levels were determined by quantitative real-time PCR using SYBR Premix Ex Taq (ABI) and GAPDH of humans and mice as internal control, respectively.

### 2.3. Immunoblotting

Immunoblotting was performed as previously described [33]. Briefly, cell or tissue samples were homogenized for dissecting in RIPA lysis buffer. The supernatant was collected after centrifuging and then incubated in 100°C for 10 min following the addition of loading buffer. After being separated by using SDS-PAGE, proteins in the gel were transferred to the polyvinylidene difluoride (PVDF) membrane. Following the incubation of the membrane with 10% fat-free milk for 1 hr membranes were respectively subjected to overnight incubation with the primary antibodies at 4°C and then incubated in horseradish peroxidase-conjugated secondary antibodies. Proteins were detected by enhanced chemiluminescence assay (Thermo Fisher Scientific).

### 2.4. Luciferase Reporter Assay

The potential promoter region of human GDF15 was inserted into the pGL3 basic plasmid, corresponding to the region of −2000 to +100 with respect to the putative transcription start site (denoted nucleotide +1). The constructed plasmids were transfected into HT29 cells to determine luciferase activities by using Dual-luciferase Assay Kit (Promega) according to the manufacturer's instructions.

### 2.5. Seahorse Analysis

FAO assays were conducted by using Seahorse XF24e extracellular flux analyzer (Agilent Technologies, Santa Clara, CA, USA) according to the instruction. In brief, CRC cells were plated in XF cell culture microplates and cultured in normal medium for 4 hours which were then replaced with a substrate-limited medium for 16 hours. FAO assay medium was added before the assay followed with the addition of XF palmitate-BSA FAO substrate. The oxygen consumption rate (OCR) was detected by Mito stress test protocols of seahorse XF24e extracellular flux analyzer.

### 2.6. Tumor Cell Invasion Assay

Invasion assays were performed using the cell invasion chamber (BD) according to the manufacturer's protocol. Briefly, HT29 and SW480 cells in serum-free medium were added to the upper chamber and medium supplemented with 2% FBS was added to the lower chamber to be subsequently subjected to normoxia or 1% O_2_ for 48 hours. The invasive tumor cells were subsequently fixed by methanol and then subjected to crystal violet staining. For quantification, the cells were counted under a microscope in randomly selected five predetermined fields (200x).

### 2.7. Establishment of GDF15 Knockdown Cells and Tumor Xenograft Mouse Model

For knockdown endogenous GDF15, shRNA designed to target human *GDF15* mRNA (shGDF15) was purchased from Invitrogen and negative control shRNA (NC) was from Genepharma. For the selection of GDF15 stable knockdown cells, the oligoduplexes synthesized based on the sequence of shGDF15 or negative control shRNA were cloned into the pRetroSuper vector (Origene). HT29 cells were transfected with shGDF15 vector (shGDF15) or control vector (NC) and then selected for 2 weeks with 2 *μ*g/ml puromycin (Sigma). For tumor xenograft mouse, HT29 cells of NC or shGDF15 were injected into the dorsal flank of female athymic nude mice (4-5 weeks old) in a laminar flow cabinet. Tumor formation in nude mice was monitored and the tumors were excised 12 days after the injection. All experiments in this study were performed in accordance with protocols approved by the Institutional Animal Care and Use Committee of Fudan University.

### 2.8. Statistical Analysis

All experiments presented in this paper have been repeated more than 3 times. Data are presented as the mean ± standard error of means (s.e.m.). Statistical analysis (GraphPad Prism 5.0) was performed using the two-tailed independent Student's *t*-test after a demonstration of homogeneity of variance with the *F* test or one-way or two-way ANOVA for more than two groups. Statistical significance was set as *p* < 0.05.

## 3. Results

### 3.1. Hypoxia-Induced ER Stress Enhances Metastasis of CRC Cells

**Figure 1:**
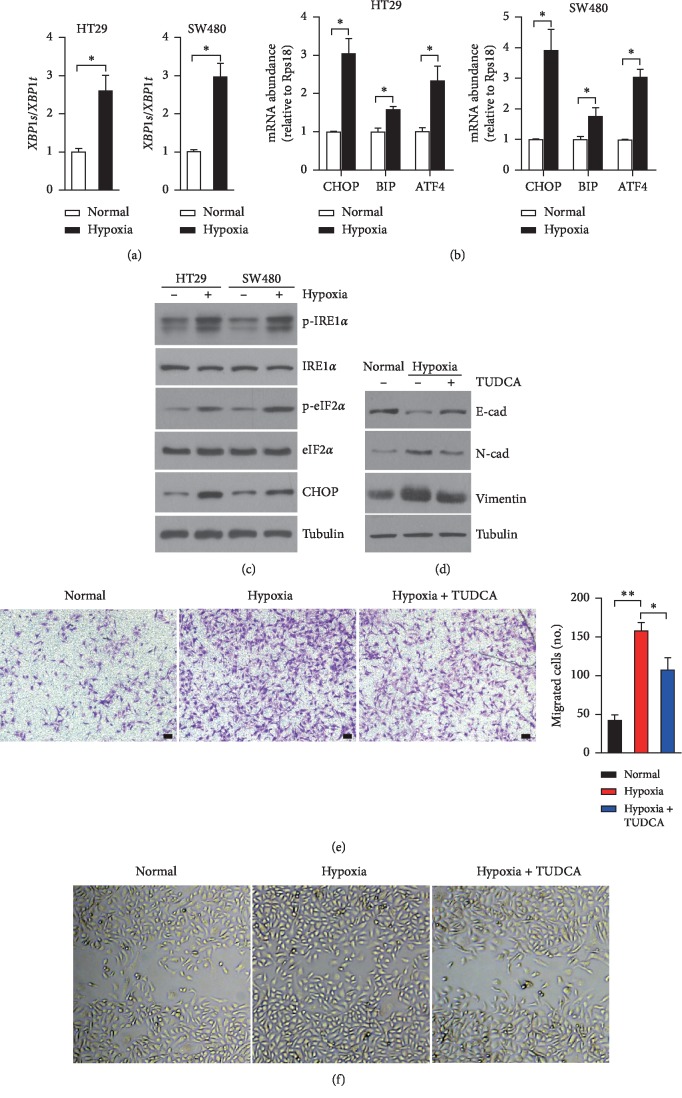
Hypoxia-induced ER stress enhances metastasis of CRC cells. (a–c) HT29 cells or SW480 cells were exposed to normal or hypoxia for 12 hours. qRT-PCR was performed to analyze splicing levels of *XBP1* (a) and mRNA abundance of UPR-associated genes (b). (c-d) Immunoblotting analysis of indicated proteins involved in UPR pathways (c) and EMT (d). (d-f) HT29 cells were exposed to normoxia or hypoxia (1% O_2_) or hypoxia with or without the presence of TUDCA (100 *μ*M). (e) Representative images of infiltrated cells detected by crystal violet staining using the Transwell system. Infiltrated cells were counted. *n* = 4 for per group. Scale bar, 50 *μ*m. (f) Representative images of wound healing assays. All data are shown as mean ± s.e.m. ^*∗*^*p* < 0.05 by unpaired two-tailed Student's *t*-test.

To explore whether hypoxia could induce ER stress, CRC cells (HT29 and SW480) were subjected to hypoxic exposure (1% O_2_). Compared to normoxia-incubated cells, both HT29 and SW480 cells exhibited extremely higher expression levels of UPR-associated genes, including spliced *XBP1* (*XBP1s/XBP1t*), *BIP*, *CHOP*, and *ATF4* (Figures 1(a) and 1(b)). Meanwhile, hypoxic exposure also exacerbated the phosphorylation levels of IRE1*α* and eIF2*α*, as well as protein levels of CHOP in both HT29 and SW480 cells, respectively (Figure 1(c)). These *in vitro* results revealed that hypoxia led by low oxygen supply causes ER stress and subsequently activates the UPR pathways in CRC cells.

To explore the physiological role of hypoxia-activated UPR pathways, tauroursodeoxycholic acid (TUDCA) was utilized to alleviate cellular ER stress in CRC cells [34]. As expected, the addition of TUDCA efficiently reduced *XBP1* splicing ([Supplementary-material supplementary-material-1]) and mRNA levels of UPR genes, including *CHOP*, *ATF4*, and *BIP* ([Supplementary-material supplementary-material-1]). As hypoxia could promote EMT and invasion of CRC cells [35], we postulated that ER stress is also involved in the metastasis of CRC cells upon hypoxic exposure. In consistency with previous studies, hypoxic treatment significantly gave rise to the protein levels of N-Cadherin and Vimentin while reduced E-cadherin in HT29 cells (Figure 1(d)) and consequently accelerated the migration and invasion of HT-29 cells (Figures 1(e) and 1(f)). Intriguing, the addition of TUDCA almost abolished the impacts on EMT-associated genes which were induced by hypoxia in HT29 cells (Figure 1(d)) and efficiently attenuated cell migration which was exacerbated by hypoxic exposure (Figures 1(e) and 1(f)). These results indicate that hypoxia-induced ER stress and UPR pathway activation are essential for CRC cell metastasis.

### 3.2. Hypoxia Gives Rise to GDF15 Expression via CHOP Signaling in CRC Cells

**Figure 2:**
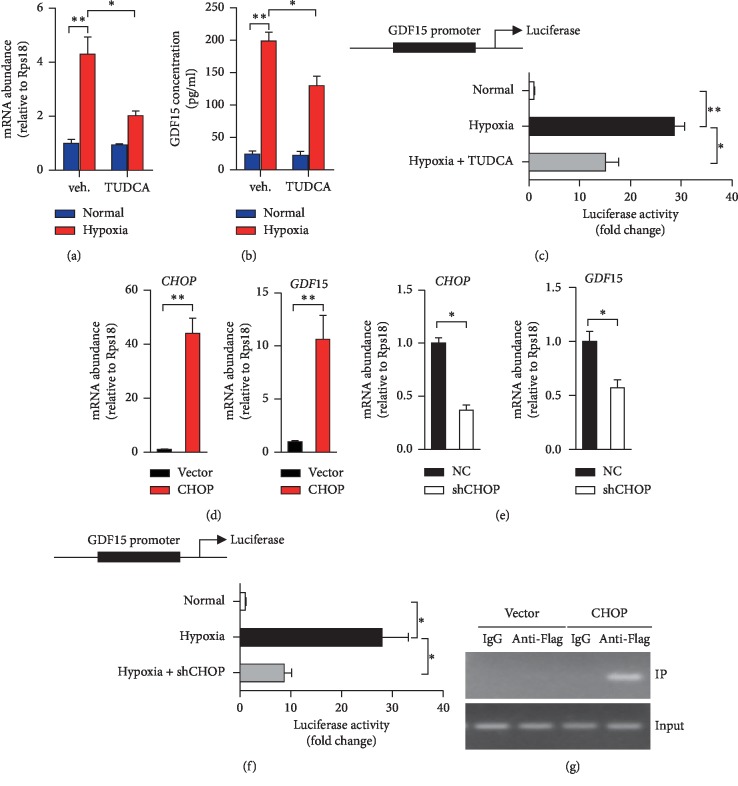
CHOP drives GDF15 expression in CRC cells upon hypoxia exposure. (a, b) HT29 cells exposed to normal air or hypoxia (1% O_2_) or hypoxia combined with TUDCA (100 *μ*M) for 12 hours. qRT-PCR analysis of *GDF15* mRNA levels (a) and ELISA analysis of GDF15 protein in cell culture (b). (c) Luciferase activity was determined in HT29 cells exposed to normal air or hypoxia or hypoxia combined with TUDCA for 24 hours. (d) qRT-PCR analysis of *CHOP* and *GDF15* in HT29 cells with the transfection of control vector or CHOP-expressing plasmid (CHOP). (e) qRT-PCR analysis of *CHOP* and *GDF15* in HT29 cells with the transfection of shRNA targeted at CHOP or negative control (NC). (f) Luciferase activity was determined in HT29 cells which were transfected with NC or shCHOP, and then exposed to normal air or hypoxia for 24 hours. (g) ChIP assays were conducted byusing IgG as control or anti-Flag antibody in cell lysis from HT29 cells transfected with plasmids of Flag-tagged CHOP or control vector. Representative image of PCR results which were performed to amplify the indicated region of the GDF15 promoter. For c and f, Luc values were presented as fold change after normalized to Renilla activity (RLU). The values of the normal group were set as “1”. All data are shown as mean ± s.e.m. ^*∗*^*p* < 0.05 or ^*∗∗*^*p* < 0.01 by unpaired two-tailed Student's *t*-test or one-way ANOVA.

Previous studies demonstrated that GDF15 is implicated as an oxygen-regulated transcript responding to hypoxia and anoxia [36, 37]. To determine whether GDF15 is regulated upon hypoxia exposure, we introduced two *ex vivo* models to mimic hypoxia, including 1% O_2_ incubation and CoCl_2_ treatment. Interestingly, *GDF15* mRNA levels and extracellular secreted GDF15 were dramatically heightened in both HT29 and SW480 cells after being incubated in 1% O_2_ (Figures 2(a) and 2(b); Figures [Supplementary-material supplementary-material-1] and [Supplementary-material supplementary-material-1]) or treated with CoCl_2_ (Figures [Supplementary-material supplementary-material-1] and [Supplementary-material supplementary-material-1]), respectively. As ER stress was reported to play an essential role in regulating *GDF15* transcription [38, 39], we utilized TUDCA to investigate whether ER stress is required for hypoxia-induced *GDF15* expression. Impressively, TUDCA treatment significantly diminished *GDF15* mRNA levels and extracellular GDF15 protein levels which were upregulated by hypoxic exposure (Figures 2(a) and 2(b)). Furthermore, luciferase reporter assays also demonstrated that the addition of TUDCA sufficiently repressed the activity of GDF15 promoter which could be highly activated by hypoxic treatment (Figure 2(c)), indicating that the activation of UPR pathways is required for regulating GDF15 transcription in CRC cells. CHOP, the critical downstream transcription factor of PERK-eIF2*α* pathway, is reported to drive GDF15 expression in skeletal muscle and hepatocytes [30, 38]. Surprisingly, ectopic expression of CHOP extremely amplified the mRNA levels of GDF15 in HT29 cells (Figure 2(d)). In contrast, the abrogation of endogenous CHOP using shRNA targeted at human *CHOP* mRNA (shCHOP) efficiently reduced GDF15 expression in HT29 cells (Figure 2(e)). Subsequently, we applied the luciferase reporter assay and chromatin immunoprecipitation (ChIP) assay to determine whether CHOP directly regulates *GDF15* transcription. The loss of CHOP strongly impaired hypoxia-activated activity of luciferase of which expression was driven by *GDF15* promoter (Figure 2(f)). Meanwhile, the chromatin of *GDF15* promoter was coimmunoprecipitated with the anti-Flag antibodies which target exogenous Flag-tagged CHOP, indicating the direct interaction between CHOP and *GDF15* promoter DNA (Figure 2(g)). Together, these results revealed that CHOP drives the transcription of GDF15 via directly binding its promoter in hypoxia-exposed CRC cells.

### 3.3. GDF15 Is Essential for Hypoxia-Promoting EMT and Invasion of CRC Cells

**Figure 3:**
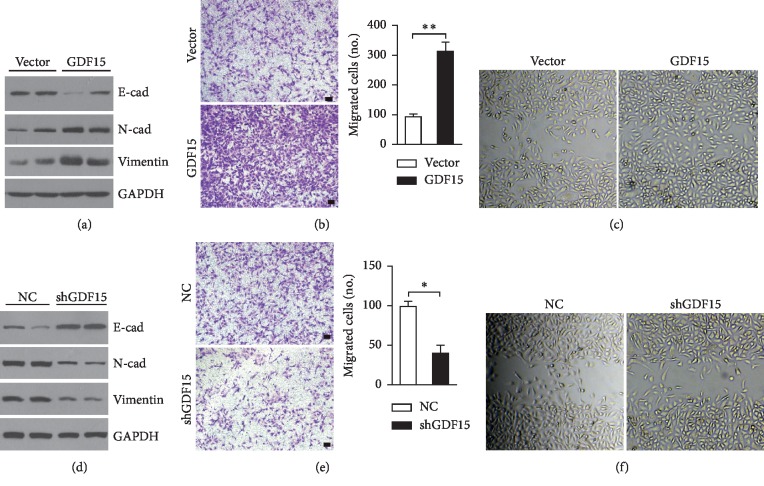
GDF15 promotes EMT and invasion of CRC cells. (a–c) HT29 cells were transfected with GDF15-expressing plasmids or control vector. (a) Immunoblotting analysis of EMT-associated protein levels. (b) Representative images of infiltrated cells detected by crystal violet staining. Infiltrated cells were counted. *N* = 4 per group. (c) Representative images of wound healing assays. (d–f) HT29 cells were transfected with the shGDF15 or negative control (NC). (d) Immunoblotting analysis of EMT-associated protein levels. (e) Representative images of infiltrated cells detected by crystal violet staining. Infiltrated cells were counted. *N* = 4 per group. (f) Representative images of wound healing assays. (b, e) scale bar, 50 *μ*m. All data are shown as mean ± s.e.m. ^*∗*^*p* < 0.05 or ^*∗∗*^*p* < 0.01 by unpaired two-tailed Student's *t*-test or one-way ANOVA.

To explore the physiological functions of upregulated GDF15 in the setting of hypoxia, we investigated the role of GDF15 in tumor cell metastasis, which is highly promoted upon hypoxia. The ectopic expression of GDF15 in HT29 cells dramatically altered protein levels of EMT-associated genes expression, including increased N-cad and Vimentin while decreased E-cad (Figure 3(a)). Moreover, GDF15-overexpressed HT29 cells also exhibited enhanced cell invasion and migration compared to the control group (Figures 3(b) and 3(c)). In accordance with the effects led by the ectopic expression, the loss of endogenous GDF15 resulted in impaired EMT (Figure 3(d)), as well as cell migration and invasion (Figures 3(e) and 3(f)). Therefore, the results showed that hypoxia-induced GDF15 is critical in regulating the metastasis of CRC cells.

### 3.4. GDF15 Enhances Fat Acid Oxidation of CRC Cells

**Figure 4:**
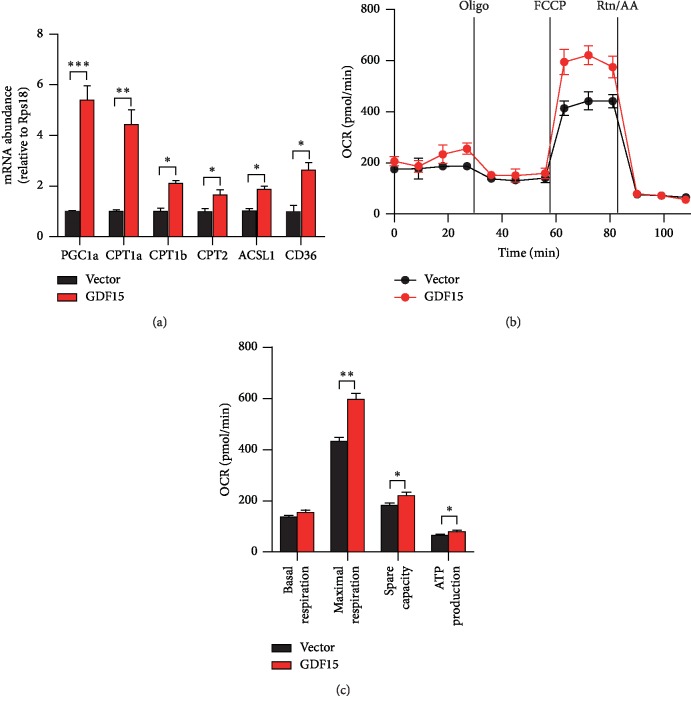
Overexpressed GDF15 enhances fat acid oxidation in CRC cells. (a) qRT-PCR analysis of FAO-associated genes expression in HT29 cells with the transfection of the control vector or GDF15-expressing plasmid (GDF15). (b, c) Analysis of mitochondrial FAO in HT29 cells by using the seahorse bioscience XF24e extracellular flux analyzer. All data are shown as mean ± s.e.m. ^*∗*^*p* < 0.05, ^*∗∗*^*p* < 0.01, ^*∗∗∗*^*p* < 0.001 by unpaired two-tailed Student's *t*-test.

A growing number of studies implicates that increased GDF15 could promote mitochondrial fat acid *β*-oxidation (FAO) [35], which is essential for metastasis of cancer cells [6, 40]. To explore whether fatty acid metabolism could be regulated by GDF15 in CRC cells, we assessed the mRNA levels of key genes involved in FAO process. Impressively, all FAO-associated genes were dramatically increased after the overexpression of GDF15 in HT29 cells (Figure 4(a)). To validate that the upregulation of the genes resulted in enhanced FAO activity, FAO assays were performed by using seahorse XF24e extracellular flux analyzer. After being subjected to starvation and then exogenous fatty acid treatment, tumor cells with GDF15 overexpression displayed robustly enhanced maximal respiration and burned much more fatty acids to supply energy (Figures 4(b) and 4(c)). Together, these results demonstrated that GDF15 promotes the usage of fatty acids via *β*-oxidation and enhances the ATP production in CRC cells.

### 3.5. Abrogation of GDF15 Represses the Development and Metastasis of Xenograft Tumor

To investigate the role of GDF15 in the growth of CRC cells, CCK8 assays were applied to evaluate cell proliferation levels. Interestingly, enforced *GDF15* expression augmented the growth rate of HT29 cells, while the silence of endogenous *GDF15* efficiently attenuated the proliferation levels of CRC cells (Figures [Supplementary-material supplementary-material-1] and [Supplementary-material supplementary-material-1]), indicating that GDF15 could promote the CRC cell growth. Next, we utilized flow cytometry by Annexin V and PI staining to assess the cell viability. Relative to control groups, the percentages of dead cells (due to both apoptosis and necrosis) were significantly reduced in the HT29 cells with overexpressed GDF15 (∼20% reduction, [Supplementary-material supplementary-material-1]) or rGDF15 treatment (∼35% reduction, [Supplementary-material supplementary-material-1]), while the percentage increased ∼9% in GDF15-deficient HT29 cells ([Supplementary-material supplementary-material-1]).

**Figure 5:**
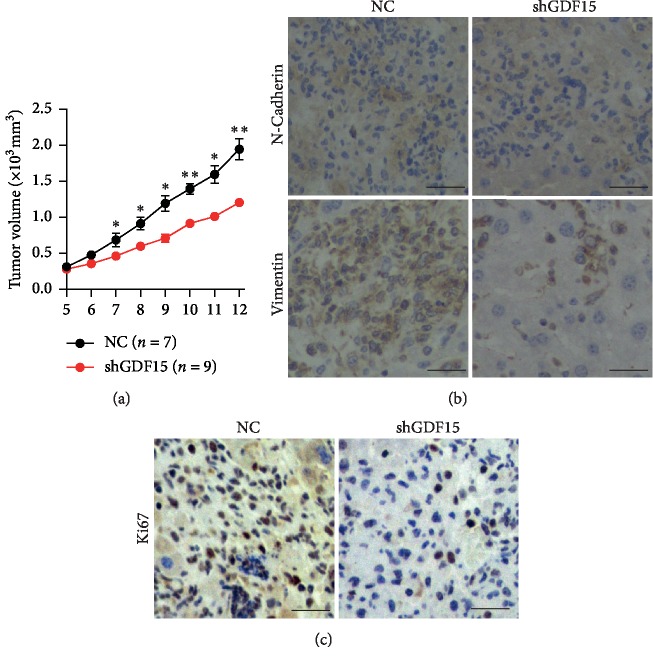
GDF15 is required for the development and metastasis of tumor *in vivo*. HT29 cells were transfected with shRNA targeted at GDF15 (shGDF15) or negative control shRNA (NC). The cells (5 × 10^6^) were implanted into nude mice at the flank to induce tumor formation and tumor size was measured. *n* = 7 for NC group and *n* = 9 for the shGDF15 group. (a) The volume of xenograft tumors. (b) Representative images of immunostaining of the sections of xenograft tumors for N-Cadherin and Vimentin. (c) Representative images of immunostaining of the sections of tumors for Ki67. (b-c) scale bar, 50 *μ*m. The data are shown as mean ± s.e.m. ^*∗*^*p* < 0.05 or ^*∗∗*^*p* < 0.01 by unpaired two-tailed Student's *t*-test.

To further elucidate the role of GDF15 in tumor progression *in vivo*, we generated HT29 cells with stably expressing shGDF15 to abrogate endogenous *GDF15* in cells. Then, HT29 cells were subcutaneously injected into the dorsal flanks of nude mice and typical tumors were observed 2 weeks later. Intriguingly, compared to the NC group, tumors of shGDF15 exhibited much smaller sizes and extreme reduction in growth rate (Figure 5(a)). Furthermore, EMT was dramatically repressed in the shGDF15-expressing tumors, in which expression levels of N-Cadherin and Vimentin were obviously reduced (Figure 5(b)), implicating the repression of tumor metastasis after GDF15 abrogation. Additionally, relative to control tumors, the shGDF15-expressing tumors displayed less Ki67-positive cells (Figure 5(c)), reflecting the impairments of cell proliferation in the shGDF15-expressing tumor. Together, these results revealed that GDF15 is required for the development and metastasis of xenograft tumors *in vivo*.

### 3.6. Overexpressed GDF15 in Tumors of Human CRC Displayed High Correlation with CHOP

**Figure 6:**
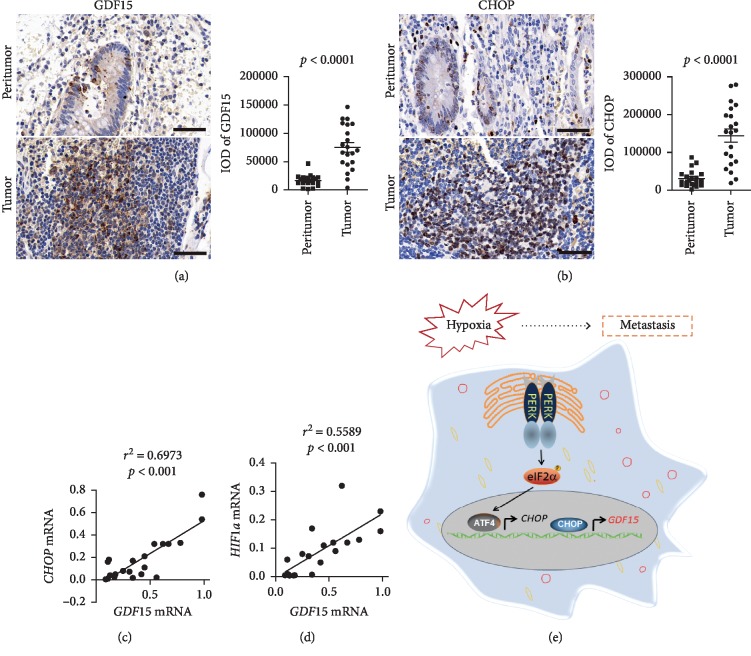
Overexpressed GDF15 displayed high correlation with CHOP in human CRC. (a-b) Representative images of IHC staining of GDF15 protein (a) and CHOP protein (b) in paired peritumor and tumor tissue slides from human CRC patients (*n* = 21 patients). Scale bar, 50 *μ*m. Results of quantitative integrated optical density (IOD) analysis were shown, respectively. *p* value is noted in the figure by unpaired two-tailed Student's *t*-test. (c-d) Linear regression analysis of mRNA levels of indicated genes in tumors of CRC patients. The coefficient of determination (*r*^2^) and *p* value are indicated. (e) Schematic model of this study. In colorectal carcinoma, hypoxic exposure causes ER stress and triggers the activation of UPR signaling pathway, including PERK-eIF2*α* branch. Elevated CHOP binds to the promoter and activates the expression of GDF15, which increases the levels of intracellular fatty acids *β*-oxidation. GDF15 promotes the metastasis as well as the growth of CRC cells.

To investigate the GDF15 expression levels in tumors of CRC patients, we analyzed the pattern of GDF15 expression in both tumor tissues and matched peritumor tissues from 21 CRC patients. The results of IHC staining exhibited that GDF15 levels of tumors were much higher than that of peritumor (Figure 6(a)), and moreover, CHOP levels also significantly increased in tumor tissues (Figure 6(b)), suggesting the positive correlation between GDF15 and CHOP. Linear regression analysis of mRNA levels in CRC tumors revealed a significantly positive correlation between *GDF15* and *CHOP* (Figure 6(c)), as well as between *GDF15* and *HIF1a* (Figure 6(d)). These data demonstrate that GDF15 is highly expressed in tumors of CRC patients and display a positive correlation with both *CHOP* and *HIF1a* in mRNA levels.

## 4. Discussion

The intrinsic properties and microenvironments of a tumor cell are critical in the dissemination and metastasis of cancer cells. In this study, we demonstrate that GDF15 plays an essential role in mediating hypoxia-initiated EMT and metastasis of colorectal cancer cells. In CRC cells, ER stress is induced upon hypoxic exposure and the alleviation of ER stress by TUDCA significantly represses hypoxia-induced EMT and metastasis of tumor cells. Further studies reveal that hypoxia gives rise to the expression of GDF15 which is driven by CHOP in the PERK-eIF2*α* signaling. Highly expressed GDF15 promotes the metastasis of CRC cells and meanwhile accelerates the growth of tumor cells (Figure 6(e)). The results of the *in vivo* study also show that GDF15 abrogation dramatically attenuates the development and metastasis of xenograft tumors derived from CRC cells.

In most solid tumors, the metastasis during which cancer cells become prone to be migratory and invasive is frequently accompanying with the hypoxic tumor microenvironment. Due to the properties of high levels of proliferation and metabolism while poor vascularization in tumors, colorectal carcinoma is often subject to many forms of stresses, including glucose deprivation, hypoxia, acidosis, and low pH value [14, 41]. In our current study, we found that hypoxia could trigger the ER stress and activate the UPR pathways, including PERK-eIF2*α* signaling (Figure 1), which is thought to be definitely implicated in tumor cell EMT [21]. Previous studies indicate that activated PERK-eIF2*α* signaling could drive the expression of GDF15 [38], which is discovered as an important mediator in promoting cell growth of prostate cancer [42] and EMT of CRC cells *in vitro* [43], and even acts as a driver in cachexia progression [44, 45]. In consistency with these studies, our current work also reveals the essential roles of hypoxia-induced GDF15 in the metastasis of CRC in *vitro* (Figure 3) and the progression of xenograft tumors *in vivo* (Figure 5). In contrary to the study of Li et al. [43], we found that besides promoting EMT of CRC cells, enhanced expression of GDF15 could also give rise to the proliferation of CRC cells ([Supplementary-material supplementary-material-1]) and is required for the growth of xenograft tumor *in vivo* (Figure 5), suggesting the multiple roles of GDF15 in CRC progression.

It is worth noting that increased GDF15 expression in CRC cells efficiently promotes the mitochondrial fatty acid *β*-oxidation and increase the production of ATP (Figure 4). Linking to the critical role of fatty acid oxidation in metastasis of tumor cells, we hypothesize that the exacerbated GDF15 regulates the EMT and metastasis of CRC cells via enhancing the utilization of fatty acids to produce more fuels for cell growth and migration. More studies are needed to fully clarify the functions and mechanisms of GDF15 in tumor progression, especially in regulating cellular metabolism of cancers.

The UPR may assist in several aspects of tumor biology, ranging from tumorigenesis, apoptotic evasion, metastasis, angiogenesis, and chemotherapy resistance [46]. Tumor progression is characterized by UPR activation induced by the challenging growth conditions associated with hypoxia [46]. Among the three branches of UPR pathways, the PERK-eIF2*α* pathway largely contributes to the growth and survival of cancer under hypoxic stress [47]. Interestingly, PERK is reported to be responsible for the activation of many angiogenic genes. However, the molecular mediator(s) underlying PERK-eIF2*α* regulating tumor cell growth and metastasis is/are not clearly uncovered. We demonstrated that upon hypoxia exposure, induced CHOP could bind to the promoter of GDF15 to activate its transcription (Figure 2), which is consistent with the previous study in skeletal muscle [30]. It is worth noting that CHOP is commonly considered as a driver for inducing cell apoptosis in most tumors including CRC [14, 48]. Our *in vitro* results, however, indicate that CHOP-activated GDF15 expression is required for the cell survival of CRC cells, suggesting a negative feedback of GDF15 in the activation PERK-eIF2*α*-CHOP signaling during hypoxia.

## 5. Conclusions

In summary, our current work reveals a novel molecular mechanism of GDF15 expression regulated by activated UPR pathway in CRC cells upon hypoxia exposure, which might serve as a promising therapeutic target for clinical treatment and drug discovery.

## Data Availability

The data used to support the findings of this study are available from the corresponding author upon request.

## Supplementary Materials

Supplementary Materials Figure S1: TUDCA alleviates hypoxia-induced ER stress in CRC cells. Figure S2: hypoxia exacerbates GDF15 expression and secretion in CRC cells. Figure S3: GDF15 accelerates cell growth and reduces cell death of CRC cells.
