# Supplementary material for: Hypoxia Induces Growth Differentiation Factor 15 to Promote the Metastasis of Colorectal Cancer via PERK-eIF2α Signaling
Source: Biomed Res Int. 2020 Jan 27;2020:5958272. doi: 10.1155/2020/5958272 (PMC7008299; doi:10.1155/2020/5958272)
Supplement: Supplementary Materials — Figure S1: TUDCA alleviates hypoxia-induced ER stress in CRC cells. Figure S2: hypoxia exacerbates GDF15 expression and secretion in CRC cells. Figure S3: GDF15 accelerates cell growth and reduces cell death of CRC cells. [file 5958272.f1.docx]

**Supplementary Materials**


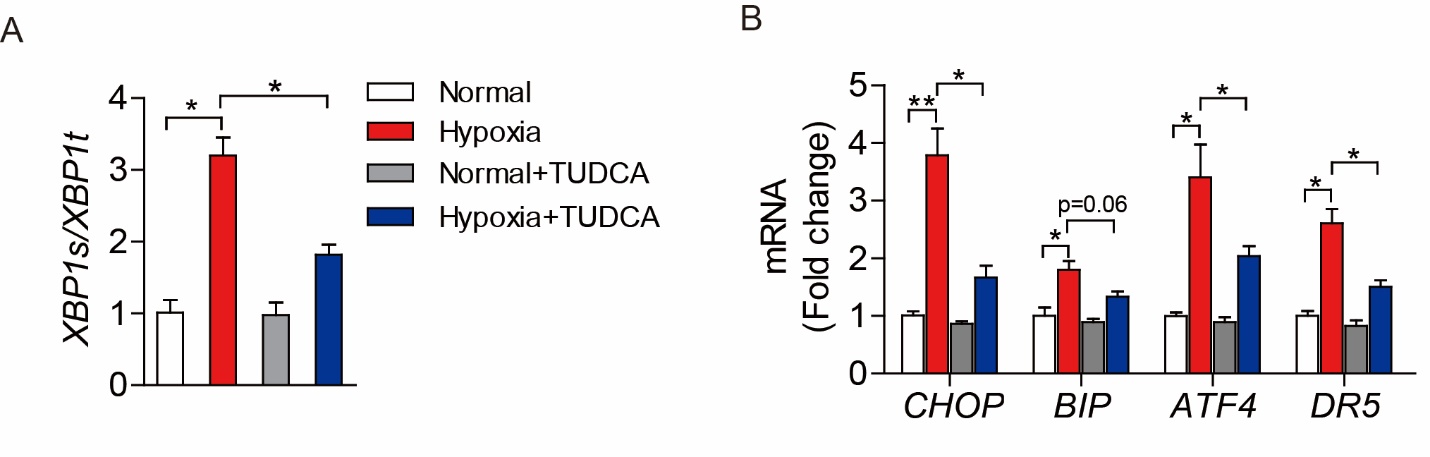


Fig.S1. TUDCA alleviates hypoxia-induced ER stress in CRC cells.

qRT-PCR analysis of XBP1 splicing (A) and mRNA levels of indicated genes (B) in HT29 cells exposed to normal air or hypoxia (1% O_2_) or hypoxia combined with TUDCA (100 μM) for 12 hours. All data are shown as mean ± s.e.m.. *, *p* <0.05 or **, *p* <0.01 by unpaired two-tailed Student’s *t*-test or one-way ANOVA.


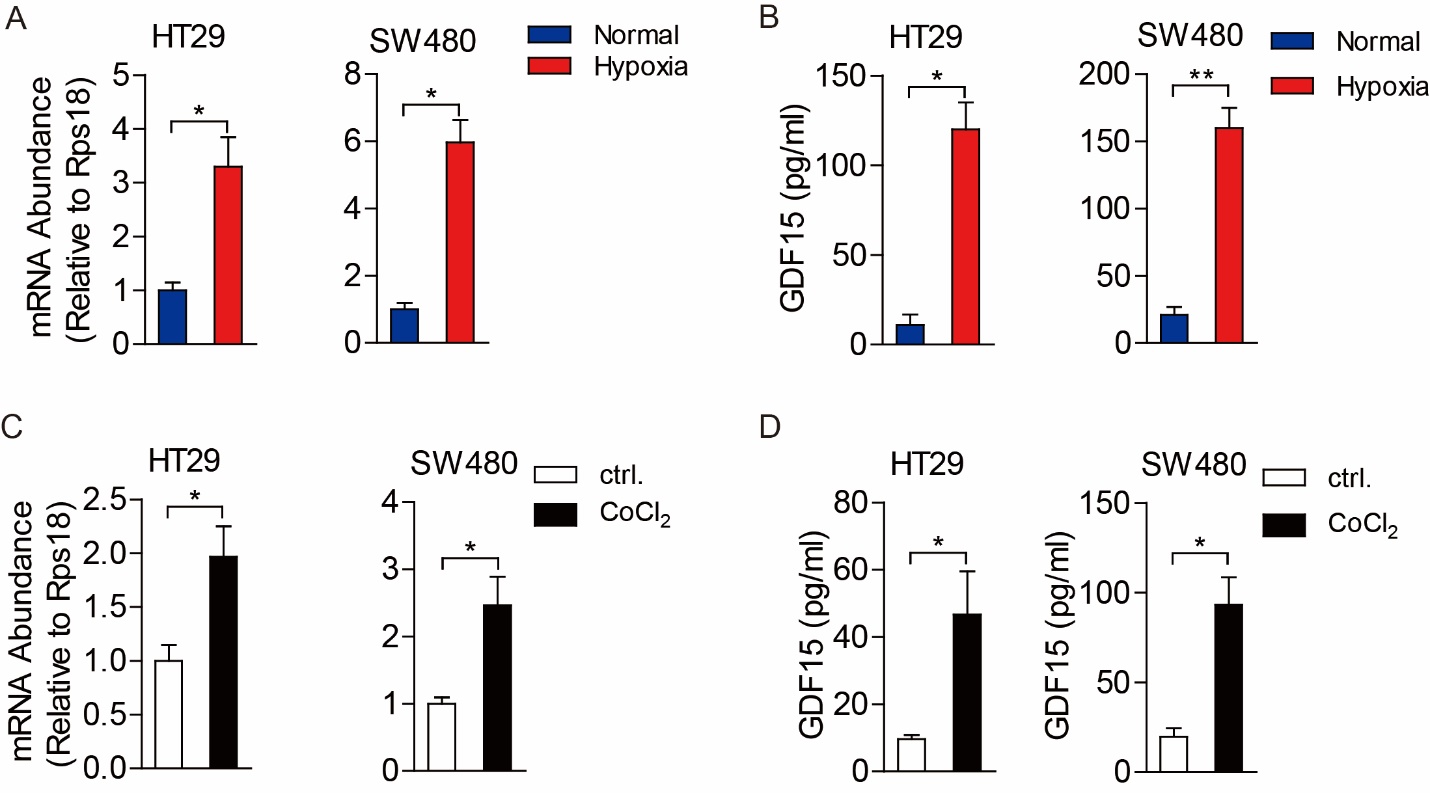


Fig.S2. Hypoxia exacerbates GDF15 expression and secretion in CRC cells.

(A-B) HT29 cells and SW480 cells were subjected to hypoxia (1% O_2_) or normal air incubation for 12 hours. A, Cells were collected to analyze *GDF15* mRNA abundance by qRT-PCR. B, extracellular GDF15 of cell-cultured medium were determined by ELISA. (C-D) HT29 cells and SW480 cells were treated by CoCl_2_ or vehicle control (ctrl.). C, Cells were collected to analyze *GDF15* mRNA abundance by qRT-PCR. D, extracellular GDF15 of cell-cultured medium were determined by ELISA. All data are shown as mean ± s.e.m.. *, *p* <0.05; **, *p* <0.01 or ***, *p* <0.001 by unpaired two-tailed Student’s *t*-test.


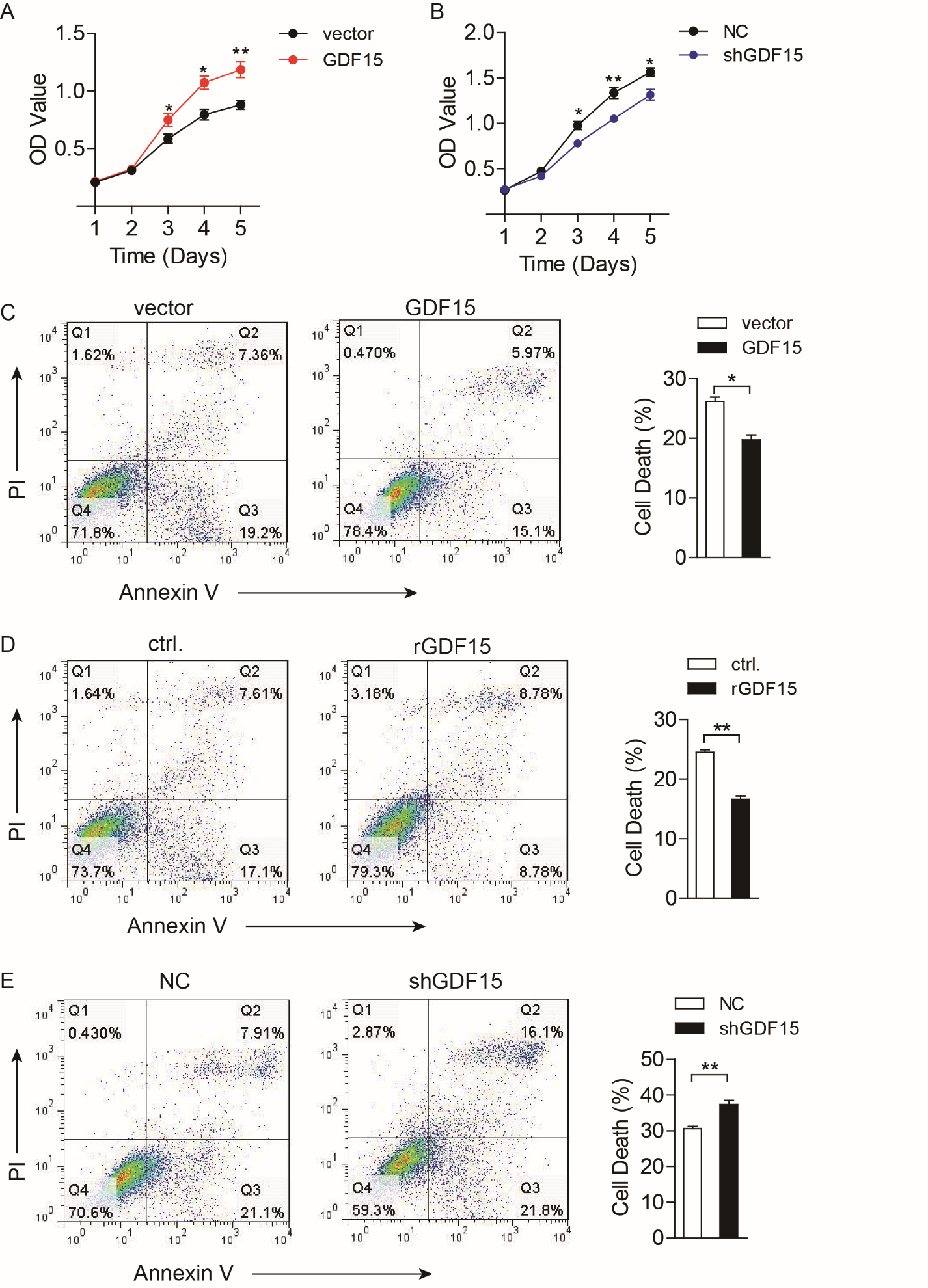


Fig. S3. GDF15 accelerates cell growth and reduces cell death of CRC cells.

A-B, MTT assays determined the cell growth rates of HT29 cell after indicated treatment

C-E, Flow cytometry assays by Annexin V-PI staining analyzed cell death of HT29 cells after indicated treatment. The percentage of dead cells were quantified by the sum of the cell percentage in Q2 and Q3 zones of each sample. A,C, Cells were transfected with vector control or GDF15-expressing plasmids. B,E, Cells were transfected with shRNA targeted at GDF15 (shGDF15) or negative control (NC). D, Cells were treated with recombinant GDF15 (rGDF15) or vehicle control (ctrl.). All data are shown as mean ± s.e.m.. *, *p* <0.05 or **, *p* <0.01 by unpaired two-tailed Student’s *t*-test or one-way ANOVA.
